# Effect of Quercetin Monoglycosides on Oxidative Stress and Gut Microbiota Diversity in Mice with Dextran Sodium Sulphate-Induced Colitis

**DOI:** 10.1155/2018/8343052

**Published:** 2018-11-12

**Authors:** Zhu Hong, Meiyu Piao

**Affiliations:** ^1^Department of Anal and Intestinal Surgery, Tianjin Union Medical Center (Nankai University Affiliated Hospital), Tianjin, China; ^2^Department of Gastroenterology and Hepatology, Tianjin Medical University, General Hospital, Tianjin 300052, China

## Abstract

The pathogenesis of inflammatory bowel disease (IBD) is linked to an intricate association of environmental, microbial, and host-related factors. This study examined the potential effects of dietary addition of two preparations from onion, one comprising quercetin aglycone alone (Q: 0.15% polyphenols, quercetin aglycone:quercetin monoglycosides, 98:2) and another comprising quercetin aglycone with monoglycosides (Q+MQ: 0.15% total polyphenols, quercetin aglycone:quercetin monoglycosides, 69:31), on dextran sodium sulphate- (DSS-) induced colitis in mice. The results revealed a significant decrease in the body weight gain of the mice with DSS-induced colitis, which was counteracted by the dietary Q or Q+MQ supplementation. Meanwhile, the oxidative stress indicated by myeloperoxidase (MPO), reduced glutathione (GSH), malondialdehyde (MDA), and serum nitrate (NO) concentrations was higher in mice with DSS-induced colitis than in the control group mice, but dietary Q or Q+MQ supplementation counteracted this trend. The colitis mice demonstrated reduced Chao1, angiotensin-converting enzyme (ACE), and Shannon indices and an increased Simpson index, but the colitis mice receiving dietary Q or Q+MQ exhibited higher Chao1, ACE, and Shannon indices and a reduced Simpson index. In conclusion, this research showed that even at a low dose, dietary Q or Q+MQ supplementation counteracts DSS-induced colitis in mice, indicating that Q or Q+MQ may be used as an adjuvant therapy for IBD patients.

## 1. Introduction

Inflammatory bowel disease (IBD) is a chronic immune system-mediated inflammatory disorder that comprises a range of conditions predominantly affecting the gastrointestinal tract [1]. These conditions include Crohn's disease, ulcerative colitis, and indeterminate colitis, a rare condition. The pathogenesis of IBD is linked to an intricate association of environmental, microbial, and host-related factors, including genetic factors; however, the precise mechanism underlying the condition remains unknown [2, 3].

Numerous contributing factors influence the pathology of IBD, including genetic, environmental, and inflammatory factors, intestinal microbiota, and oxidative stress conditions [4–6]. Oxidative stress is associated with protein, lipid, and DNA damage due to oxidation reactions and is thus considered to be responsible for the development of many pathological disorders or exacerbation of symptoms, such as inflammation [7–9]. Recently, the significant role of intestinal microbiota in the pathophysiology of IBD has been emphasized. Most studies have reported reduced diversity of intestinal microbiota in IBD patients [10, 11], with reduced Firmicutes population and increased Proteobacteria population being the most commonly observed variations [3, 11]. The reduced diversity of intestinal microbiota observed in IBD patients is considered to be predominantly caused by a reduction in Firmicute diversity.

Although therapeutic drugs, such as steroids, immunomodulators, and antibodies, are available to treat IBD, their administration is limited due to their serious side-effects, including infection, malignancy, and general health concerns [12, 13]. Several studies that have investigated nutritional controls for IBD, with particular focus on polyphenols, have reported that a variety of polyphenols can be used as potential therapeutic approaches for IBD [14].

Onions are known to contain high levels of polyphenols, the most abundant of which is quercetin [15, 16]. Quercetin mostly occurs in the glycoside form, although the free aglycone form is also reasonably common [16]. Studies have shown that quercetin exhibits antihypertensive, antidepressant, and anti-inflammatory properties by participating in several intracellular biochemical mechanisms [17]. However, different polyphenolic compounds within the diet may have different effects on the host physiology, either locally within the intestinal tract or on other tissues [18]. The present study investigated the protective effects of dietary addition of two preparations from onion, one rich in quercetin aglycone alone (Q) and another containing quercetin aglycone with monoglycosides (Q+MQ), on dextran sodium sulphate- (DSS-) induced colitis in mice.

## 2. Materials and Methods

### 2.1. Preparation of Quercetin and Quercetin Monoglycosides

Quercetin and quercetin monoglycosides were prepared according to the method described by Katarzyna Grzelak-Błaszczyk et al. [18]. In brief, raw onions (50 kg) were subjected to extraction by immersing them in 50% aqueous ethanol solution (1:5) in a dynamic large-scale laboratory extractor at 35°C for 6 h. The extract was then evaporated to obtain 5% ethanol extract, followed by purification in a column filled with Amberlite XAD 1600N. Gravitational elution was performed at a flow rate of 1.6–7 ml/min using 10%–70% ethanol solutions. Of these, 10%–40% fractions were rejected, and only 50%–70% fractions were used to obtain a fraction of quercetin monoglycosides with quercetin aglycone and a separate fraction containing a high concentration of quercetin aglycone. The fractions were concentrated to 10–12°Bx and freeze-dried under 0.26 mbar vacuum for 48 h. The two resulting preparations were (1) quercetin aglycone with quercetin monoglycosides (Q+MQ) and (2) quercetin aglycone (Q). Polyphenol concentrations were then confirmed using ultra-high-performance liquid chromatography as described by Katarzyna Grzelak-Błaszczyk et al. [18].

The obtained onion polyphenol preparations had different polyphenol compositions. The Q+MQ preparation comprised 42.5% polyphenols, the main polyphenol of which was free quercetin (68.4% of the total polyphenols) and remaining polyphenols were quercetin monoglycosides (31.6%). The Q preparation contained a high concentration of polyphenols of approximately 73.7%, comprising quercetin aglycone as the predominant polyphenol (97.8% of the total polyphenols) with small quantities of quercetin monoglycosides.

### 2.2. Experimental Design

The experiment was performed in accordance with the Guidelines for Animal Welfare and Experimental Protocol and was approved by the Animal Care and Use Committee of Nankai University Affiliated Hospital. Forty female ICR mice, each weighing approximately 21 g, were used in this experiment. The mice were kept in separate cages maintained at 24°C ± 3°C, 49%  ± 5% humidity, and a 12-h light–dark cycle.

The 40 female mice were randomly assigned to four equal groups: the control group (CTRL), which received a basal rodent diet; a DSS treatment group (DSS); a QUE group (QUE); and a QMQ group (QMQ). All groups received a basal rodent diet [19]; however, the control and DSS group diets were devoid of polyphenols, whereas the QUE group diet was supplemented with 0.21% Q preparation comprising 0.15% polyphenols (quercetin aglycone:quercetin monoglycosides, 98:2) and the QMQ diet was supplemented with 0.36% Q+MQ preparation comprising 0.15% polyphenols (quercetin aglycone:quercetin monoglycosides, 69:31). The mice were allowed free access to their respective diets and clean water for 7 days. On the eighth day, the CTRL group mice were permitted free access to clean water, but the DSS, QUE, and QMQ group mice received drinking water containing 3% DSS solution (Kayon Biological Technology Co. Ltd.) for 7 days to induce colitis [19]. Each mouse was weighed at the start and end of the experimental procedure. At the end of this experiment, each mouse was sacrificed for measuring the colon length and weight. Further histological evaluations were performed as described by Jie Yin et al. [2]. Furthermore, colon contents from each mouse were collected and immediately frozen in liquid nitrogen for storage at −70°C until use.

### 2.3. Assessment of Serum Oxidative Stress

Myeloperoxidase (MPO), reduced glutathione (GSH), and malondialdehyde (MDA) concentrations were assessed using commercial kits (Nanjing Jiancheng Bioengineering Institute, Nanjing, Jiangsu, China) in accordance with the manufacturer's instructions. Serum nitrate (NO) concentrations were approximated by measuring the absorbance of the azo-product resulting from the reaction between nitrite, sulfonamide, and N-(1-napthyl)ethylenediamine at 543 nm, in accordance with the methods described by Miranda et al. [20].

### 2.4. Microbial DNA Isolation and Sequencing

Microbial DNA was purified from mouse colon contents using a QIAamp DNA Stool Mini Kit (Qiagen) in accordance with the manufacturer's instructions. Microbial community diversity and composition for each sample was assessed and calculated by sequencing the V4 region of 16S rDNA. The total DNA was amplified with primers 515F (5′-GTGCCAGCMGCCGCGGTAA-3′) and 806R (5′-GGACTACHVGGGTWTCTAAT-3′) as described previously [21]. The DNA samples were then forwarded to a commercial service provider (Novogene, Beijing, China) for sequencing on an Illumina MiSeq platform in accordance with the manufacturer's guidelines [22]. Raw data were acquired prior to screening and assembly using the QIIME [23] and FLASH [22] software packages. Sequencing reads were assigned to samples according to the barcodes. Reads flagged as chimeric were eliminated to obtain an effective sequence collection for every sample. The QIIME software package and UPARSE pipeline were used to evaluate these effective sequences and identify operational taxonomic units (OTUs). Subsequently, the UCLUST algorithm was used to cluster sequences with a 97% identity into the same OTU. Each OTU was allotted to a specific taxonomic level using the RDP Classifier [24].

### 2.5. Statistical Analysis

All data are presented as the mean and standard error of the mean (SEM). Statistical analysis was performed using SPSS 22.0 software (SPSS, Chicago, IL). The results were statistically evaluated using one-way analysis of variance, and significant differences between groups were determined using Duncan's multiple range test with statistical significance set at* P* < 0.05.

## 3. Results

**Figure 1:**
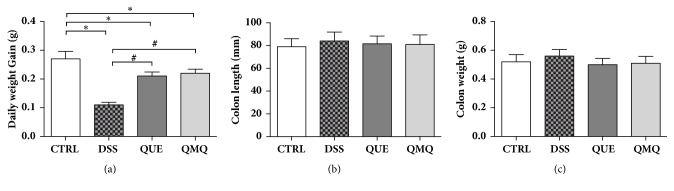
Illustration showing the effect of administering Q or Q+MQ on (a) average daily weight gain, (b) colon length, and (c) colon weight following DSS treatment. *∗* indicates significant difference compared with the CTRL group (*P* < 0.05); # indicates significant difference compared with the DSS group (*P* < 0.05) (all,* n* = 10).

The results demonstrated a significant reduction (*P* < 0.05) in the average daily gain in the DSS group, but the dietary Q and Q+MQ in the QUE and QMQ groups, respectively, seemed to significantly counteract this DSS-induced growth suppression (*P* < 0.05) (Figure 1(a)). In addition, the length and weight of the colon showed no significant differences between the four groups (Figures 1(b) and 1(c)).

**Figure 2:**
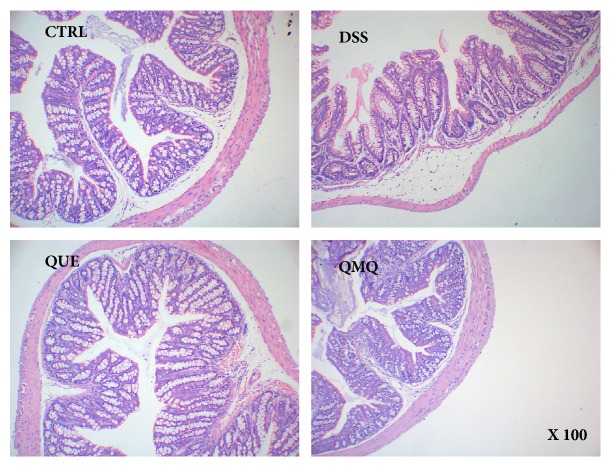
Histological evaluation of the hematoxylin and eosin-stained sections of the colon (×100) from the four groups (all, *n* = 10).

Figure 2 presents the results of histological evaluation of the colon. No histological injury was observed in the colon of the CTRL group, whereas mice exposed to DSS in the DSS, QUE, and QMQ groups showed scattered villi, neutrophil infiltration, and desquamation in the colon. The histological status of the QUE and QMQ groups was much better than that of the DSS group.

**Figure 3:**
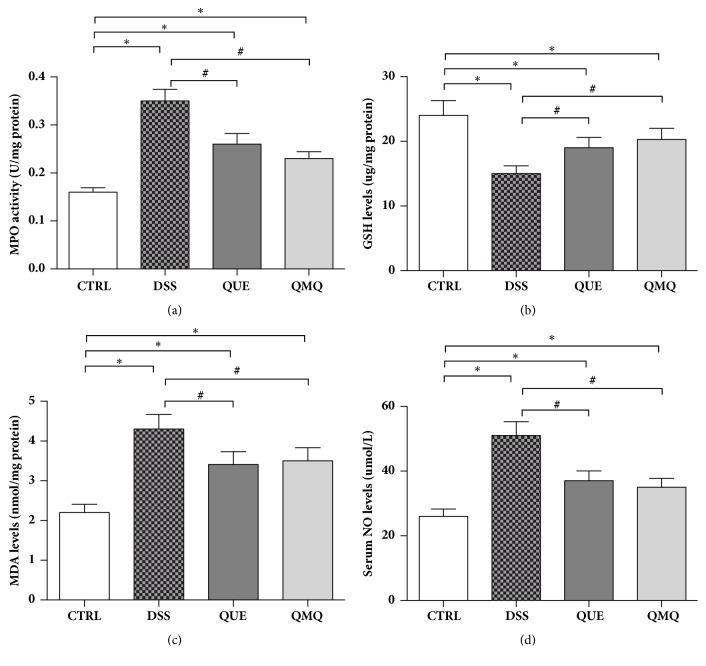
Effect of quercetin on (a) MPO, (b) reduced GSH, (c) MDA, and (d) serum NO concentrations in DSS-induced IBD in mice (*n* = 10). *∗* indicates significance at* P* < 0.05 relative to the CTRL group; # indicates significance at* P* < 0.05 relative to the DSS group.

The MPO activity significantly increased (*P* < 0.05) in DSS-treated mice compared with that in the CTRL group mice. However, Q and Q+MQ administration in the QUE and QMQ groups, respectively, significantly counteracted this increase in the MPO activity (P < 0.05) compared with that in the DSS group (Figure 3(a)). Further, the GSH level significantly decreased (*P* < 0.05) in DSS-treated mice compared with that in the CTRL group mice. However, Q and Q+MQ administration in the QUE and QMQ groups, respectively, significantly counteracted this reduction in the GSH activity (*P* < 0.05) compared with that in the DSS group (Figure 3(b)). Moreover, the MDA and serum NO concentrations significantly increased (*P* < 0.05) in DSS-treated mice compared with that in the CTRL group mice. However, Q and Q+MQ administration in the QUE and QMQ groups, respectively, significantly counteracted this increase in MDA and serum NO concentrations (*P* < 0.05) compared with that in the DSS group (Figures 3(c) and 3(d)).

To evaluate the effects of Q and Q+MQ on microbial communities, the V4 region of the 16S rRNA gene was sequenced. In total, 1,064,746 sequences (25,038 ± 1432 per sample) were obtained, including 38135, 39198, 37542, and 39011 raw reads obtained from samples in the CTRL, DSS, QUE, and QMQ groups, respectively. Following trimming, assembly, and quality filtering, 4563 OTUs were identified, and the indices of community richness and diversity are presented in Table 1. Compared with the CTRL group, the DSS, QUE, and QMQ groups demonstrated reduced Chao1, angiotensin-converting enzyme (ACE), and Shannon indices and an increased Simpson index (*P* < 0.05). Conversely, compared with the DSS group, the QUE and QMQ groups exhibited higher Chao1, ACE, and Shannon indices (*P* < 0.05) and a reduced Simpson index (*P* < 0.05). Nevertheless, no significant alpha diversity was detected in the microbiota in the QUE and QMQ groups.

**Figure 4:**
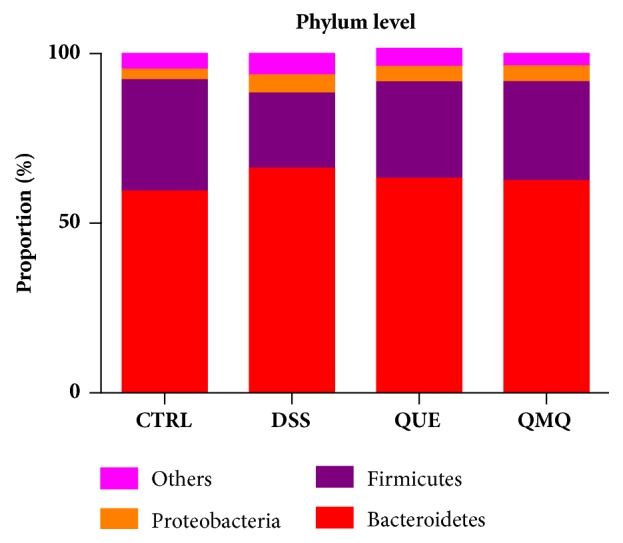
Effect of quercetin on colonic microbiota at the phylum level in mice with DSS-induced IBD (*n* = 10).

Intestinal microbiota diversity was evaluated using taxon-dependent analysis. Six phyla were detected in the colonic contents across all four groups. The three most predominant phyla across all four groups were Bacteroidetes, Firmicutes, and Proteobacteria. The proportion of Bacteroidetes, Firmicutes, and Proteobacteria populations were 59.45%, 32.87%, and 3.05% of the total microbiota, respectively, in the CTRL group; 66.19%, 22.16%, and 5.34%, respectively, in the DSS group; 63.24%, 28.42%, and 4.54%, respectively, in the QUE group; and 62.53%, 29.15%, and 4.68%, respectively, in the QMQ group (Figure 4).

**Figure 5:**
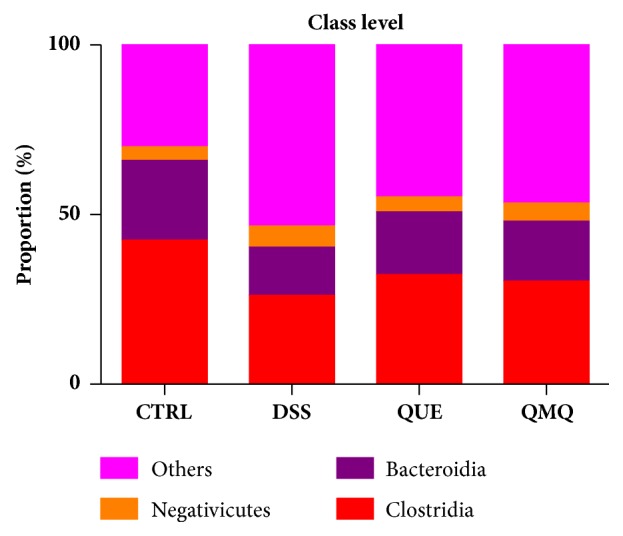
Effect of quercetin on colonic microbiota at the class level in mice with DSS-induced IBD (*n* = 10).

The three most predominant classes across all four groups were Clostridia, Bacteroidia, and Negativicutes, in addition to small quantities of Bacilli, Gammaproteobacteria, and Erysipelotrichia. The proportions of Clostridia, Bacteroidia, and Negativicutes populations were 42.48%, 23.49%, and 3.95%, respectively, in the CTRL group; 26.14%, 14.22%, and 6.24%, respectively, in the DSS group; 32.33%, 18.43%, and 4.46%, respectively, in the QUE group; and 30.37%, 17.63%, and 5.36%, respectively, in the QMQ group (Figure 5).

## 4. Discussion

Preparations from onion were demonstrated to be good sources of polyphenols. The extraction and purification procedures delivered preparations with high-purity polyphenols of varying concentrations. This study showed that dietary Q and Q+MQ were effective in reducing DSS-induced colitis in mice, which is thought to manifest via reduction in oxidative stress and colonic microbiota diversity. To the best of our knowledge, only a limited number of studies have focused on investigating natural compounds capable of reversing experimental colitis. In the current study, 3% DSS was used to induce acute colitis so that the effect of Q and Q+MQ on DSS-induced colitis could be investigated. Weight loss is the traditional clinical index for IBD [25], and the results of this study showed that DSS-containing water caused neutrophil infiltration and scattered villi in the colon and a significant decrease in average daily weight gain, consistent with the results of previous studies [2, 26]. However, both Q and Q+MQ ameliorated these changes.

Increased oxidative stress is associated with mucosal inflammation in ulcerative colitis, and it may be a contributing factor to the progression to malignancy associated with this disorder [27, 28]. The biological tests performed in the current study validated the antioxidant effects of the preparations, which included free quercetin in addition to free Q and Q+MQ, on the DSS-induced colitis in mice. Compared with the CTRL group mice, a significant increase (*P* < 0.05) was observed in the MPO, MDA, and NO concentrations in mice receiving DSS water. However, compared with the DSS group, both Q and Q+MQ significantly counteracted this increase in MPO, MDA, and NO concentrations (*P* < 0.05) in the QUE and QMQ groups, respectively. The MPO activity in the colonic mucosa is used as an inflammatory response marker and is linearly correlated with neutrophil infiltration [29]. Several studies have shown that MPO is present at high concentrations in IBD patients [29, 30]. Inflammation is associated with the recruitment and activation of mucosal phagocytic leukocytes, which release high levels of reactive oxygen species (ROS), e.g., superoxide anion [31]. Such unregulated overproduction of ROS may overwhelm protective mechanisms, leading to cellular oxidative damage [32].

Both the Shannon and Simpson indices indicate diversity within any community and are affected by parameters of species richness and species evenness within that sample community. With respect to same species richness, the greater the uniformity of the species within the community, the greater the diversity of the microbial community. Consistent with previous findings [3, 11], our findings indicated that DSS treatment reduces colonic microbiota diversity. The human intestines contain large populations of microorganisms, and extensive alterations in the microbial composition and the regular function of the intestinal microbiota have been associated with IBD [10, 11]. Typically, the Firmicutes-to-Bacteroidetes ratio is a key indicator of intestinal microbiota composition, and lower Firmicutes-to-Bacteroidetes ratios may be associated with weight loss because reduced Firmicutes populations reduce the additional energy provided to the host by them via fermentation of polysaccharides to short-chain fatty acids [25]. The present study demonstrated that DSS treatment reduces Firmicutes population and increases Proteobacteria population but that Q or Q+MQ supplementation can ameliorate these effects. The results also indicated that dietary Q or Q+MQ supplementation may alter the outcomes of microbiota-associated disorders. In IBD patients, intestinal microbiota dysbiosis is frequently noted. This condition is frequently characterized by an increased abundance of facultative anaerobic bacteria (e.g., Enterobacteriaceae and Bacilli) and simultaneous reduction of obligate anaerobic bacteria from the Bacteroidia and Clostridia classes [33, 34].

In conclusion, this study demonstrated that DSS treatment for 7 days results in signs of colitis, including growth reduction and colonic inflammation. Dietary Q or Q+MQ supplementation, even at a low dose, was found to counteract DSS-induced colitis in mice. Because quercetin is a ubiquitous phytochemical present in a variety of fruits and vegetables, implementation of a quercetin-supplemented diet for treating IBD represents a feasible approach with considerable potential. Thus, Q or Q+MQ may be administered as an adjuvant therapy for IBD patients. Additional mechanistic and toxicity investigations to examine the efficacy of this phytochemical for IBD prevention and treatment are recommended.

## Figures and Tables

**Table 1:** Alpha diversity indices of the colonic contents of mice with DSS-induced IBD.

Items	CTRL	DSS	QUE	QMQ	SEM	*P*
OTU	1239	1028	1144	1122	98.4	0.37
Chao1	1189.23	998.45^*∗*^	1104.81^*∗*#^	1096.45^*∗*#^	45.6	0.039
ACE	1230.14	1057.48^*∗*^	1155.47^*∗*#^	1136.74^*∗*#^	76.3	0.045
Shannon	5.39	4.81^*∗*^	5.07^*∗*#^	5.11^*∗*#^	0.212	0.035
Simpson	0.84	0.93^*∗*#^	0.89^*∗*#^	0.88^*∗*#^	0.064	0.041

*∗* indicates significance at *P* < 0.05 relative to the CTRL group; # indicates significance at *P* < 0.05 relative to the DSS group.

## Data Availability

The data used to support the findings of this study are available from the corresponding author upon request.

## References

[1] Clarke K., Chintanaboina J. (2018). Allergic and Immunologic Perspectives of Inflammatory Bowel Disease. *Clinical Reviews in Allergy & Immunology*.

[2] Yin J., Wu M., Duan J. (2015). Pyrrolidine Dithiocarbamate Inhibits NF-KappaB Activation and Upregulates the Expression of Gpx1, Gpx4, Occludin, and ZO-1 in DSS-Induced Colitis. *Applied Biochemistry and Biotechnology*.

[3] Zhu D., Ma Y., Ding S., Jiang H., Fang J. (2018). Effects of melatonin on intestinal microbiota and oxidative stress in colitis mice. *BioMed Research International*.

[4] Matsuoka K., Kanai T. (2015). The gut microbiota and inflammatory bowel disease. *Seminars in Immunopathology*.

[5] Khor B., Gardet A., Xavier R. J. (2011). Genetics and pathogenesis of inflammatory bowel disease. *Nature*.

[6] Ananthakrishnan A. N. (2013). Environmental risk factors for inflammatory bowel disease. *Gastroenterol Hepatol*.

[7] Salim S., Sarraj N., Taneja M., Saha K., Tejada-Simon M. V., Chugh G. (2010). Moderate treadmill exercise prevents oxidative stress-induced anxiety-like behavior in rats. *Behavioural Brain Research*.

[8] Salim S. (2014). Oxidative stress and psychological disorders. *Current Neuropharmacology*.

[9] Hu S.-Y., Jia X.-Y., Li J.-N. (2016). T cell infiltration is associated with kidney injury in patients with anti-glomerular basement membrane disease. *Science China-Life Sciences*.

[10] Tong M., Li X., Parfrey L. W. (2013). A modular organization of the human intestinal mucosal microbiota and its association with inflammatory bowel disease. *PLoS ONE*.

[11] Willing B. P., Dicksved J., Halfvarson J. (2010). A pyrosequencing study in twins shows that gastrointestinal microbial profiles vary with inflammatory bowel disease phenotypes. *Gastroenterology*.

[12] McLean L. P., Cross R. K. (2014). Adverse events in IBD: to stop or continue immune suppressant and biologic treatment. *Expert Review of Gastroenterology & Hepatology*.

[13] Colombel J.-F., Sands B. E., Rutgeerts P. (2016). The safety of vedolizumab for ulcerative colitis and Crohn's disease. *Gut*.

[14] Ding S., Jiang H., Fang J. (2018). Regulation of immune function by polyphenols. *Journal of Immunology Research*.

[15] Panche A. N., Diwan A. D., Chandra S. R. (2016). Flavonoids: an overview. *Journal of Nutritional Science*.

[16] Chen S., Jiang H., Wu X., Fang J. (2016). Therapeutic effects of quercetin on inflammation, obesity, and type 2 diabetes. *Mediators of Inflammation*.

[17] Su Q., Peng M., Zhang Y. (2016). Quercetin induces bladder cancer cells apoptosis by activation of AMPK signaling pathway. *American Journal of Cancer Research*.

[18] Grzelak-Błaszczyk K., Milala J., Kosmala M. (2018). Onion quercetin monoglycosides alter microbial activity and increase antioxidant capacity. *The Journal of Nutritional Biochemistry*.

[19] Ren W., Yin J., Wu M. (2014). Serum amino acids profile and the beneficial effects of L-arginine or L-glutamine supplementation in dextran sulfate sodium colitis. *PLoS ONE*.

[20] Hollman P. C. H., van Trijp J. M. P., Buysman M. N. C. P. (1997). Relative bioavailability of the antioxidant flavonoid quercetin from various foods in man. *FEBS Letters*.

[21] Kong X.-F., Ji Y.-J., Li H.-W. (2016). Colonic luminal microbiota and bacterial metabolite composition in pregnant Huanjiang mini-pigs: Effects of food composition at different times of pregnancy. *Scientific Reports*.

[22] Magoč T., Salzberg S. L. (2011). FLASH: fast length adjustment of short reads to improve genome assemblies. *Bioinformatics*.

[23] Caporaso J. G., Kuczynski J., Stombaugh J. (2010). QIIME allows analysis of high-throughput community sequencing data. *Nature Methods*.

[24] Wang Q., Garrity G. M., Tiedje J. M., Cole J. R. (2007). Naïve Bayesian classifier for rapid assignment of rRNA sequences into the new bacterial taxonomy. *Applied and Environmental Microbiology*.

[25] Chen S., Wang M., Yin L. (2018). Effects of dietary tryptophan supplementation in the acetic acid-induced colitis mouse model. *Food & Function*.

[26] Kim J. J., Shajib M. S., Manocha M. M., Khan W. I. (2012). Investigating intestinal inflammation in DSS-induced model of IBD. *Journal of Visualized Experiments*.

[27] Ullman T. A., Itzkowitz S. H. (2011). Intestinal inflammation and cancer. *Gastroenterology*.

[28] Bhattacharyya A., Chattopadhyay R., Mitra S., Crowe S. E. (2014). Oxidative stress: an essential factor in the pathogenesis of gastrointestinal mucosal diseases. *Physiological Reviews*.

[29] Iba Y., Sugimoto Y., Kamei C., Masukawa T. (2003). Possible role of mucosal mast cells in the recovery process of colitis induced by dextran sulfate sodium in rats. *International Immunopharmacology*.

[30] Arab H. H., Salama S. A., Eid A. H., Omar H. A., Arafa E.-S. A., Maghrabi I. A. (2014). Camel's milk ameliorates TNBS-induced colitis in rats via downregulation of inflammatory cytokines and oxidative stress. *Food and Chemical Toxicology*.

[31] Zhu H., Li Y. R. (2012). Oxidative stress and redox signaling mechanisms of inflammatory bowel disease: updated experimental and clinical evidence. *Experimental Biology and Medicine*.

[32] Pavlick K. P., Laroux F. S., Fuseler J. (2002). Role of reactive metabolites of oxygen and nitrogen in inflammatory bowel disease. *Free Radical Biology & Medicine*.

[33] Minamoto Y., Otoni C. C., Steelman S. M. (2015). Alteration of the fecal microbiota and serum metabolite profiles in dogs with idiopathic inflammatory bowel disease. *Gut Microbes*.

[34] Stecher B. (2015). The roles of inflammation, nutrient availability and the commensal microbiota in enteric pathogen infection. *Microbiology Spectrum*.

